# Procyanidin Suppresses Tumor Growth by Activating the B-Cell MAPK Pathway through Remodulation of the Gut Microbiota and Metabolites in Hepatocellular Carcinoma

**DOI:** 10.7150/ijbs.113217

**Published:** 2026-01-01

**Authors:** Ran Huo, Chen-Zheng Gu, Yang Liu, Zi-Xian Wei, Te Liu, Jie Zhu, Lin Ding, Yu Liu, Chu-Yu Wang, Yi-Ni Li, Xin-Yi He, Wen-Jing Yang, Bei-Li Wang, Yun-Wei Wei, Wei Guo

**Affiliations:** 1 Department of Laboratory Medicine, Zhongshan Hospital, Fudan University, Shanghai, China.; 2 Department of Laboratory Medicine, Shanghai Geriatric Medical Center, Shanghai, China.; 3 Department of Laboratory Medicine, Xiamen Branch, Zhongshan Hospital, Fudan University, Xiamen, China.; 4 Department of Laboratory Medicine, Wusong Branch, Zhongshan Hospital, Fudan University, Shanghai, China.; 5 Cancer Center, Shanghai Zhongshan Hospital, Fudan University, Shanghai, China.; 6 Department of Clinical Laboratory, Clinical Oncology School of Fujian Medical University, Fujian Cancer Hospital, Fuzhou, China.; 7 Shanghai Geriatric Institute of Chinese Medicine, Shanghai University of Traditional Chinese Medicine, Shanghai, China.; 8 Department of Pancreatic and Gastrointestinal Surgery Division, Ningbo No.2 Hospital, Ningbo, China.; 9 Ningbo Key Laboratory of Intestinal Microecology and Human Major Diseases, Ningbo, China.; 10 Department of Hepatobiliary and Pancreatic Surgery, Qunli Branch, the First Afiliated Hospital of Harbin Medical University, Harbin, China.

**Keywords:** hepatocellular carcinoma, procyanidin, gut microbiota, 5-hydroxytryptophan, B cells, MAPK pathway

## Abstract

The mortality of hepatocellular carcinoma (HCC) is high. Plant-derived bioactive compounds have emerged as potential therapies for HCC. Procyanidin (PAC) has been shown to possess immune-modulating and anti-tumor properties. However, the role and mechanism of total PAC in treating HCC remain unclear. We established subcutaneous and orthotopic HCC mouse models to assess the effect of PAC on tumor growth. Multi-omics analyses and *in vitro* experiments were conducted to investigate the changes in the gut microbiota, related-metabolites, and the tumor microenvironment (TME). 16S rDNA sequencing revealed that PAC could reshape the gut microbiota, notably increasing *Lactobacillus murinus* abundance. Furthermore, transplantation of *Lactobacillus murinus* reduced tumor volumes in mice. Single-cell RNA sequencing showed upregulation of the MAPK pathway in B cells within the TME. Metabolomic analysis suggests that 5-Hydroxytryptophan (5-HTP) derived from *Lactobacillus murinus* was significantly increased in B cells from mesenteric lymph nodes (MLNs) in the PAC-treated group. *In vitro* experiments revealed that 5-HTP could significantly upregulate the MAPK pathway in B cells. Additionally, 5-HTP-educated B cells could activate IFN-γ^+^CD8^+^T cells through B cell-T cell interactions, indicating that 5-HTP is a key metabolite in the therapeutic effect of PAC. Finally, feeding 5-HTP to HCC mice reduced tumor volume, upregulated the MAPK pathway in B cells from MLNs, and activated IFN-γ^+^CD8^+^T cells in the TME. PAC reshapes the gut microbiota and metabolites, upregulates the MAPK pathway in B cells from MLNs, and activates CD8^+^T cells in the TME through the gut-liver axis, thereby inhibiting HCC progression.

## Introduction

Hepatocellular carcinoma (HCC) is the third most common cause of cancer-related death globally, with almost 900,000 new cases each year 1. Despite the availability of various treatment options, the prognosis for HCC patients remains bleak, with a 5-year relative survival rate of only 18%, emphasizing the critical demand for innovative therapeutics. Studies have revealed the anti-tumor effects of various compounds derived from medicinal plants. One class of polyphenol compounds, the procyanidin (PAC), is found in the seeds of apples, pears, grapes, and black beans. Regularly consumed in the human diet, PAC offers essential nutrients and have been shown to inhibit multiple cancer types, including colorectal, gastric, prostate, and breast cancers 2, 3. Different forms of PAC reportedly exhibit distinct anti-cancer activities. For example, PAC C1 exerts significant anti-tumor effects by inducing DNA damage, inhibiting cell cycle progression, and activating apoptotic pathways, thereby suppressing cancer cell proliferation. Additionally, PAC C1 has been shown to inhibit tumor growth and metastasis in colorectal cancer 4. Total PAC, comprising various monomeric and polymeric forms, exhibits broader biological activities, suppressing cancer cell growth and migration while modulating signaling pathways and inhibiting angiogenesis to exert anti-cancer effects. A-type and B-type PAC has been less extensively studied, but they are thought to exhibit anti-cancer mechanisms similar to total PAC, primarily involving free radical scavenging, inflammation suppression, and apoptosis induction. Although A-, B-, and C-type PAC each possesses distinct anti-cancer properties, the diverse and synergistic effects of total PAC have made it a promising focus for anti-cancer research. However, gaps remain in our understanding of PAC bioavailability and its precise mechanisms of action, warranting further investigation 5, 6. Recent studies highlight their broad influence, demonstrating direct effects on immune cell signaling, pathogen interactions, and integrity of the intestinal mucosal barrier, as well as modulation of immune cell recruitment and inflammatory cytokine release, with a critical role in restoring gut homeostasis 7, 8. Studies have also been found to reduce the intestinal ratio of Firmicutes to Bacteroidetes, increasing the abundance of *Ruminococcus* and* Akkermansia*, and improving insulin resistance, suggesting a potential for reshaping the gut microbiota 9, 10. Emerging evidence also indicates that the gut microbiota plays a significant role in the development of HCC through immune response mediated by the gut-liver axis 11. Notably, gut microbes can exert anti-tumor effects by restoring host immune cell function. However, the mechanism by which PAC influence the growth of liver cancer through the gut microbiota is unclear.

In this study, we aimed to investigate how PAC suppresses HCC tumor growth and modulate immune cells through the gut microbiota. Two mouse models of liver cancer were established and treated with total PAC to explore its effects on tumor growth. The impact of PAC on the gut microbiota was then explored using 16S rDNA sequencing. Subsequently, the mechanisms by which PAC reshapes the gut microbiota and influences tumor growth were investigated through *in vivo* and *in vitro* experiments and analyses, including single-cell RNA sequencing, microbiota transplantation, and metabolomics. Our study findings reveal new therapeutic mechanisms for PAC in HCC, providing a theoretical foundation and practical insights for its clinical application potential in treating HCC (Supplemental fig. 1).

## Materials and Methods

### Animals

Male BALB/c mice, aged five weeks (Weitonglihua Co., Ltd., Beijing), were accommodated at the Laboratory Animal Center, Fudan University. All mice were maintained in a specific pathogen-free (SPF) environment with controlled conditions: a temperature of 22±2°C, relative humidity at 55±10%, and a 12-hour alternating light/dark cycle. After a-week-long period of acclimatization to these conditions, all experimental protocols were conducted in strict accordance with the ethical guidelines approved by the Animal Ethics Committee of Shanghai Medical College, Fudan University (No.202105002S).

### Cell culture

All cells were cultured in a humidified incubator at 37°C with 5% CO₂. Specifically, H22 cells (Cell Bank of the Chinese Academy of Sciences, China) were maintained in a mixed medium consisting of 10% fetal bovine serum (Gibco, USA), 1% penicillin/streptomycin (Gibco, USA), and 89% RPMI-1640 medium (Gibco, USA). Hepa 1-6 cells (Cell Bank of the Chinese Academy of Sciences, China) were cultured under similar conditions but with 89% DMEM medium (Gibco, USA). B and T cells were cultured in a medium with 10% fetal bovine serum, 1% penicillin/streptomycin, and 89% RPMI-1640 medium, supplemented with 10 ng/mL IL-3 (B cells, MCE, USA) or 100 U/mL IL-2 (T cells, MCE, USA) for 24~48 hours before being passaged at a 1:2 ratio. To simulate the mesenteric lymph node (MLN) environment, B and T cells were co-cultured at a 3:7 by cell number 12, and co-cultured with adherent Hepa1-6 cells at a 1:1 ratio for 0, 24, 48, and 72 hours. The cells were washed with PBS three times, and CCK-8 was used to measure cell viability of HCC. For the supernatant of *Lactobacillus murinus* (*L. murinus*), the culture broth was centrifuged at 5000g for 10 minutes, and the supernatant was filtered through a 0.22 µm filter to obtain the *L. murinus* broth culture medium (BCM). SCH772984 (MCE, USA), 5-hydroxytryptophan (5-HTP, MCE, USA), and DMSO (MCE, USA) were utilized in the cellular experiments.

### Animal model

H22 cells were collected, centrifuged, and washed twice with PBS (Beyotime, China). The cells were resuspended in PBS, adjusting the concentration to 2×10^7^/mL, and 0.1 mL was subcutaneously injected into each mouse to induce subcutaneous tumor formation 13. Calipers were used to measure the size of the subcutaneous tumors in mice, and tumor volume was calculated using the formula: Volume (mm³) = 0.5 × Length × Width^2^. For the mouse orthotopic liver cancer model, the following steps were performed: similarly, prepared H22 cells were adjusted to 1×10^7^/mL with PBS. Recipient BALB/c mice were shaved, anesthetized, disinfected with povidone-iodine, and incisions were made in the skin and peritoneum. The liver was exposed using a cotton swab soaked in PBS. A syringe was used to slowly inject 0.1 mL of the tumor cell suspension subcapsularly into the liver. Then, bleeding was controlled with dry cotton swabs, and the abdominal wall was sutured in layers with 0#5 sutures (Johnson & Johnson, USA). The incision was disinfected, and gentamicin (Rongjiarun Co., China) was injected. Mice were kept warm during anesthesia recovery. Mice were gavaged daily with 100 mg/kg/mouse PAC 14 (MCE, USA) or 100 mg/kg/mouse 5-HTP 15 for 24 days. ① BALB/c male mice were adaptively fed from day -14 to day 7, i) Subcutaneous liver cancer models or orthotopic liver cancer models were established using mice liver tumor H22 cells from day -7 to day 0, the mice were treated with 250 μL PAC (100 mg/kg/mouse, dissolved by PBS) in the treated group or 250 μL PBS in the control group once daily by gavage from day 0 to day 24, and harvested. ii) Orthotopic liver cancer models were established in tumor-bearing mice (T) group and tumor-bearing mice treated with PAC (T+P) group, using mice liver tumor H22 cells from day -7 to day 0, the mice were treated with 250 μL PAC (100 mg/kg/mouse, dissolved by PBS) in the T+P group or 250 μL PBS in the T group and tumor-free group once daily by gavage from day 0 to day 24, and harvested. ② BALB/c male mice were adaptively fed from day -28 to day -21, i) The mice in the ABX group and the *L.m* (Microbial Culture Collection Center, Chinese Academy of Sciences, China) group were given ABX (1 mg/mL ampicillin, 1 mg/mL neomycin, 1 mg/mL metronidazole, and 0.5 mg/mL vancomycin; Aladdin, China) in their drinking water (dissolved by PBS), and the mice in the control group received PBS drinking water from day -21 to day -7. On day 0, the mice were subjected to orthotopic liver cancer modeling using H22 cells. Then, from day 0 to day 24, the mice in the ABX group and the Con group received 250 μL PBS by gavage once daily, and the *L.m* group received 250 μL of an *L.m* suspension washed and resuspended in PBS (1× 10^8^ ~2 × 10^8^ CFU) by gavage once daily. ii) From day -21 to day 0, mice assigned to the ABX and 5-HTP groups received the ABX in their drinking water, whereas control mice were given PBS. On day 0, all animals underwent orthotopic H22 cells. Thereafter, daily gavage was performed from day 0 to day 24 as follows: ABX and control groups received 250 μL PBS, and the 5-HTP group received 250 μL 5-HTP at 100 mg/kg.

### Microbiota transplantation

*Lactobacillus murinus* was cultured in M17 broth medium (Ruichu Bio, China) at 37 °C under anaerobic conditions with sterile paraffin oil. The bacterial growth was monitored daily. Each mouse was orally gavaged with 1 × 10^8^~2 × 10^8^ CFU the bacterial strain suspended in 250 μL PBS once a day.

### 16S rDNA analysis

Fecal samples were collected for genomic DNA extraction using QIAGEN kits (Germantown, MD, USA), followed by assessment of DNA concentration and purity using NanoDrop 2000 spectrophotometer (Thermo Fisher Scientific, USA). The entire procedure was conducted on ice. Briefly, the V3~V4 region was PCR-amplified using 338F (ACTCCTACGGGAGGCAGCAG) and 806R (GGACTACHVGGGTWTCTAAT) primers (ABI, Los Angeles, USA), followed by library construction and sequencing. Optimization of sequence extraction involved removal of singleton non-redundant sequences to reduce computational redundancy during analysis (http://drive5.com/usearch/manual/singletons.html). The optimized sequences were grouped into operational taxonomic units (OTUs) with a 97% similarity threshold using Usearch 11. The most abundant sequence within each OTU was chosen as its representative sequence. Taxonomic classification of the OTU representative sequences was performed using RDP Classifier against the 16S rRNA gene database (Silva v138) with a confidence threshold of 0.7.

To analyze the similarities or differences in community structures among various sample groups, hierarchical clustering of sample community distance matrices was executed using the UPGMA (Unweighted Pair-group Method with Arithmetic Mean) algorithm, based on β-diversity distance matrices. UPGMA dendrograms provided a visual representation of the similarity or dissimilarity in community compositions across diverse environmental samples. Significant differences in taxonomic groups, based on abundance, were determined using non-parametric Kruskal-Wallis and unpaired Wilcoxon rank-sum tests.

### LC-MS/MS analysis

We initially placed the samples into centrifuge tubes for metabolite extraction, which is facilitated by the addition of methanol and an internal standard. The mixture is then subjected to grinding with a cryogenic tissue grinder at -10°C and a frequency of 50 Hz for a duration of 6 minutes. This is immediately followed by ultrasonic extraction at a temperature of 5°C and a frequency of 40 kHz for 30 minutes. Afterward, the samples are allowed to stand at -20 °C for 30 minutes and then centrifuged at 4°C with a force of 13000 g for 15 minutes in preparation for subsequent analysis. To ensure the reliability of the results, a quality control (QC) sample is introduced after every 5 to 15 samples, which is created by blending equal volumes of all sample metabolites.

For the LC-MS/MS analysis, the samples are run on the Thermo Fisher Scientific UHPLC-Q Exactive HF-X system. Post-analysis, raw data from the LC-MS are processed using Progenesis QI software. This software performs a series of operations including baseline filtering, peak identification, integration, retention time correction, and peak alignment, culminating in the generation of a comprehensive data matrix that captures retention time, mass-to-charge ratio, and peak intensity. Metabolite identification is achieved by matching the MS and MS/MS spectral data with entries in the HMDB and Metlin databases.

The data matrix, once preprocessed, is analyzed further through principal component analysis (PCA) and orthogonal partial least squares discriminant analysis (OPLS-DA), utilizing the ropls package in R. The identification of significantly different metabolites is based on the variable importance in projection (VIP) scores derived from the OPLS-DA model, along with p-values obtained from the student's t-test. A threshold of VIP > 1 and P < 0.05 is applied to determine statistical significance.

### Single-cell RNA sequencing (scRNA-seq)

Tissue samples are placed in a sterile petri dish, rinsed and agitated with 10 mL of pre-chilled PBS, digested, filtered through a 70 μm sieve, and centrifuged for 5 minutes, after which the supernatant is discarded. Red blood cells are lysed, and the remaining cells are counted and assessed for viability. The single-cell suspension is subjected to quality control and counting, ensuring a cell viability of over 80%. The qualified cells are washed and resuspended to prepare an appropriate cell concentration of 700~1200 cells/μL. Subsequently, GEM (Gel Bead in Emulsion) generation and barcoding, Post GEM-RT purification, cDNA amplification, library construction, and quantification are performed. The constructed library is sequenced using the Illumina Xplus platform with a PE150 sequencing mode, aiming for a sequencing depth of 20,000 reads per cell or higher.

Further quality control filtering is performed using Seurat v4 software to remove doublets, dead cells, and cell debris. Dimensionality reduction and clustering analysis, gene identification, and visualization are conducted, followed by cell type identification and related analyses using SingleR software.

### RT-qPCR

Total RNA was extracted from tissue samples using the Total RNA Extraction Kit (Promega, USA), adhering closely to the protocol. The tissue was quickly transferred to an RNase-free tube, and RNA lysis buffer was added according to the sample volume, then placed in an ice bath. A tissue homogenizer was used to disrupt the cells. Based on the starting amount and lysis buffer volume for different samples, RNA dilution buffer was added, mixed with a pipette, and incubated at room temperature for 3 to 5 minutes. According to the kit instructions, the extracted RNA was stored at -80°C. Reverse transcription was conducted immediately using the GoScript Reverse Transcription Mix (Promega, USA) following this protocol: 25 °C for 5 minutes, 42 °C for 60 minutes, 70 °C for 15 minutes, and 4 °C for one cycle. Quantitative PCR was performed with a qPCR instrument (Tailong, China) and GoTaq qPCR Master Mix (Promega, USA). The cycling conditions were: 1 cycle of polymerase activation at 95 °C for 2 minutes, followed by 40 cycles of denaturation at 95 °C for 15 seconds, and annealing and extension at 60 °C for 1 minute. Primer sequences were synthesized by Bosun Biotech Co., Ltd., Shanghai. The primer sequences were as follows: for *Sos1*, forward: 5′-CAAGTTCACCCTACTCTTGAGTC-3′, reverse: 5′-GGCATCAGCTATTGCCCAC-3′; for *Kras*, forward: 5′-CAAGAGCGCCTTGACGATACA-3′, reverse: 5′-CCAAGAGACAGGTTTCTCCATC-3′; for *Mapk1*, forward: 5′-GGTTGTTCCCAAATGCTGACT-3′, reverse: 5′-CAACTTCAATCCTCTTGTGAGGG-3′; for *GAPDH*, forward: 5′-AGGTCGGTGTGAACGGATTTG-3′, reverse: 5′-GGGGTCGTTGATGGCAACA-3′. The mRNA levels were normalized to GAPDH mRNA and expressed relative to the control group, and relative quantities were calculated.

### Western blot

Fresh tissues were thoroughly rinsed with PBS and then finely chopped into small pieces. These tissue fragments were subsequently treated with a digestive enzyme mixture in 50 mL advanced DMEM/F12 medium (Gibco, USA). The mixture contained 50 mg collagenase type IV, 25 mg hyaluronidase, and 5 mg deoxyribonuclease (Gibco, USA). The sample was incubated at 37°C with gentle agitation until a homogenous cell suspension was achieved. The suspension was then passed through a 0.22 μm filter to yield a single-cell solution. Total protein was extracted from this solution using RIPA buffer (Beyotime, China), and the protein concentration was determined with an Enhanced BCA Protein Assay Kit (Beyotime, China). For protein separation, a 10% SDS-PAGE gel (Beyotime, China) was utilized. Electrophoresis was conducted at 80 V for 30 minutes, followed by a higher voltage of 120 V for 60 minutes. The separated proteins were then transferred onto a 0.22 μm PVDF membrane (Millipore, Germany). The membrane was blocked using QuickBlock Blocking Buffer (Beyotime, China) for 30 minutes with shaking and subsequently washed with TBST buffer (Sango Biotech, China) containing Tween-20 (Sango Biotech, China) for 15 minutes at room temperature. Primary antibodies, diluted at a ratio of 1:1000 (CST, USA), were applied overnight at 4°C with gentle shaking. After washing with TBST, the membrane was incubated with HRP-conjugated secondary antibodies, also diluted at 1:1000 (Beyotime, China) for 60 minutes at room temperature. The final step involved multiple washes of the membrane, followed by visualization of the protein bands using an ECL detection reagent (Beyotime, China).

### Tissue staining or immunohistochemistry

Tissue samples were first isolated and then cut into small pieces for quick fixation in a 4% paraformaldehyde solution, which was left to act overnight. The tissues, now fixed with formaldehyde, were embedded in paraffin and subsequently sectioned. The sections were prepared for staining using either Hematoxylin and Eosin (H&E, Beyotime, China) or a Ki67 antibody (CST, USA).

For the H&E staining process, the sections underwent a series of incubations in xylene baths (xylene I and II) for 10 minutes each. This was followed by a graded ethanol series (absolute ethanol I and II, then 95%, 90%, 80%, and 70% ethanol), with each step lasting for 5 minutes. The staining procedure involved a 5-minute application of Harris hematoxylin, a brief differentiation in 1% hydrochloric acid alcohol, and subsequent rinsing. The blueing step was executed in 0.6% ammonia water, followed by another rinse. The final step in H&E staining was a 2-minute application of eosin solution (Beyotime, China). After staining, the sections were dehydrated, cleared, and mounted with neutral resin.

In the case of immunohistochemistry, the paraffin-embedded sections were first dewaxed and subjected to antigen retrieval in a buffer at 95 °C for 10 minutes. Blocking of non-specific binding sites was performed using a 10% serum solution at room temperature for 10 minutes. The sections were then incubated with specific primary antibodies overnight at 4 °C. Following this, a biotinylated secondary antibody solution was applied and incubated at 37 °C for 30 minutes. The stained sections were examined using a pathology scanner (Leica, Germany).

### Isolation of mononuclear cells

The isolation of mononuclear cells from tissues was performed using Lympholyte-M Cell Separation Media (Cedarlane, Canada), strictly adhering to the manufacturer's instructions. In brief, tissues such as spleens and lymph nodes were minced and gently triturated in an appropriate PBS to prepare a single-cell suspension. This suspension was then passed through a 70µm filter and slowly layered onto Lympholyte-M Cell Separation Media in a 1:1 ratio. The mixture was centrifuged at 800g for 30 minutes at 20°C, with an acceleration rate of 2 and no brake. Following centrifugation, the interphase layer containing the mononuclear cells was aspirated, and the cells underwent red blood cell lysis using a lysis buffer (Beyotime, China). The cells were then washed twice with PBS and centrifuged at 400g for 10 minutes before being resuspended. Finally, total B cells and total T cells were separately isolated using the Pan-B or Pan-T Cell Isolation Kit (Miltenyi Biotec, Germany), respectively, through a magnetic separator for subsequent experiments.

### Flow cytometry

Cells were gently transferred to a 1.5-mL microtube with a PBS-rinsed pipette, pelleted by centrifugation, and resuspended in 1 mL D-PBS. Fixable Viability Stain 510 (BD, USA) was added, and the suspension was incubated for 15 min at room temperature (RT) in the dark. After a second centrifugation, the supernatant was removed and the pellet was taken up in stain buffer (BD, USA). A third spin was performed, the buffer was aspirated to dryness, and the cells were left in the residual droplet. Non-specific binding was blocked with Purified Rat Anti-Mouse CD16/CD32 (BD, USA) in the dark. PerCP-Cy5.5 Hamster Anti-Mouse CD3 and FITC Rat Anti-Mouse CD8a (BD, USA) were then added; staining proceeded for 15 min at RT. Cells were washed with 1 mL PBS, centrifuged, and the supernatant discarded. The pellet was resuspended in 250 μL 1× TF Fix/Perm Buffer (BD, USA) and incubated at 4 °C for 20 min. PE Rat Anti-Mouse IFN-γ (BD, USA) was introduced, followed by a 30-min incubation at 4 °C in the dark. Finally, cells were washed once and analyzed on a flow cytometer (Sony, Japan).

### Cell counting kit-8 assay

Cell viability was evaluated using the Cell Counting Kit-8 (CCK-8, Beyotime, China). In brief, 4000 cells were seeded per well in a 96-well plate. CCK-8 reagent was then added to each well containing 100 μL of culture medium and incubated for 1 hour at 37°C with 5% CO₂. Absorbance was measured at 450 nm using a microplate reader (Sunrise Ltd., China).

### ELISA

IgG, IgA, LPS, and 5-HTP levels were quantified using ELISA kits (Elabscience, China). Samples and standards were added to the appropriate wells of the plate. The plate was then covered, incubated, and washed. Biotinylated antibody working solution was introduced, the plate covered and incubated at 37 °C, followed by washing. Each well was filled with wash buffer, soaked, washed, and blotted dry, repeating three times. Enzyme conjugate working solution was added, the plate was covered and incubated, and the washing step repeated five times. Substrate solution was introduced, the plate covered and incubated at 37 °C in the dark. The reaction was stopped with stop solution, and the optical density (OD) was immediately measured at 450 nm using a microplate reader (Bio-Rad, USA).

### Statistical analysis

Data were analyzed using SPSS software (version 20.0, USA). Flow cytometry data were plotted using FlowJo (version 10.8.1, USA). Graphs were generated using GraphPad Prism (version 8.0, USA) and R software (version 3.3.1, USA). *P*< 0.05 was considered statistically significant.

## Results

### Procyanidin suppresses tumor growth in both subcutaneous and orthotopic liver cancer mouse models

**Figure 1:**
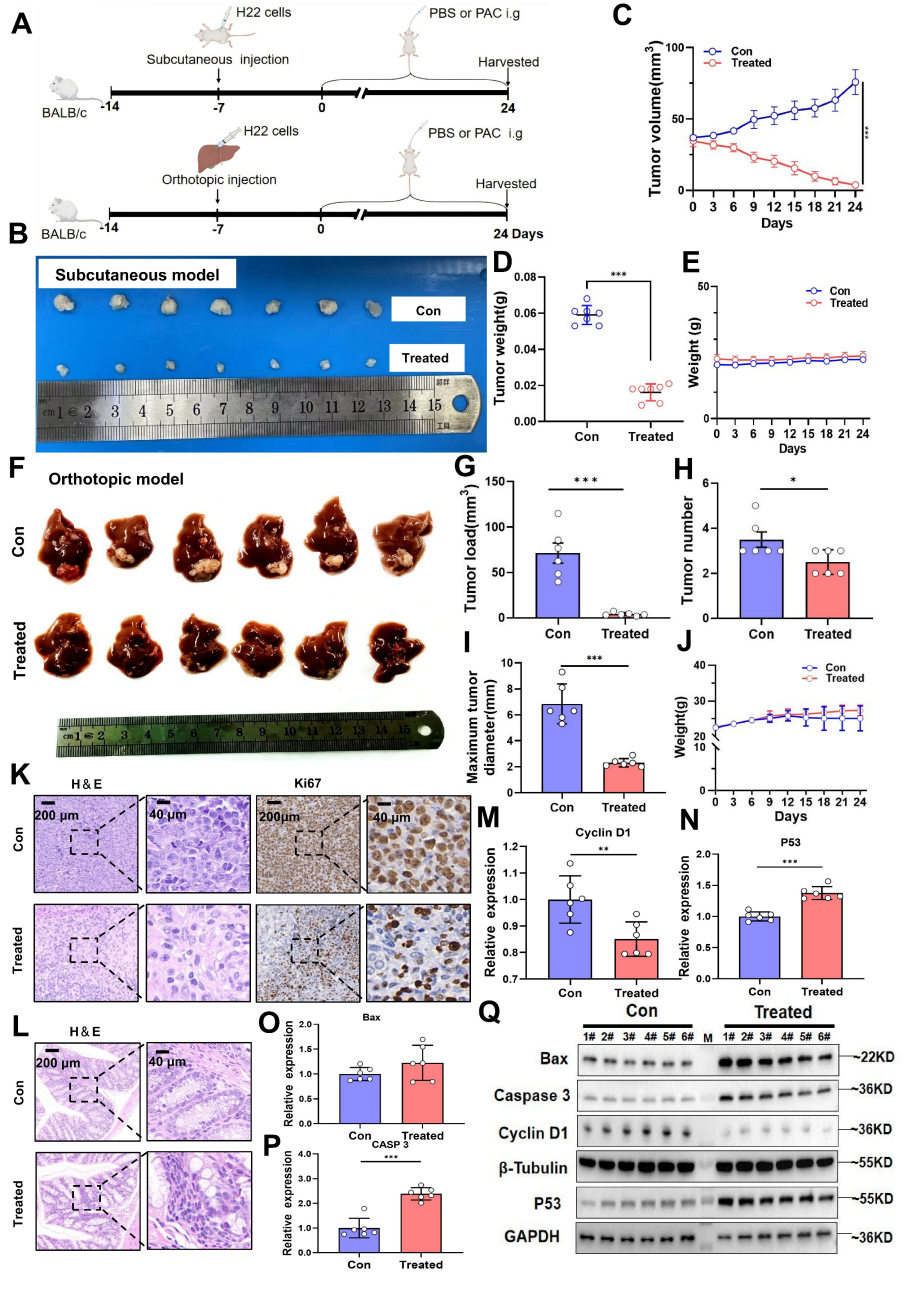
** Procyanidin suppresses tumor growth in both subcutaneous and orthotopic liver cancer mouse models.** A. Flowchart of the animal experiment. B. Photographs of subcutaneous tumors. C. Line graph depicting tumor volume changes. D. Bar graph showing tumor weight. E. Line graph of body weight changes in mice. n=7 per group. F. Images of orthotopic liver tumors. G. Bar graph of tumor load. H. Bar graph of tumor number. I. Bar graph of maximum tumor diameter. J. Line graph of body weight changes in mice. K. H&E and Ki67 staining images of tumors. L. H&E staining of the colon. M~P. Bar graphs showing relative gene expression levels, n=6 per group. Q. Western blot images of proteins. n=6 per group. M, ladder. Significance was calculated using t test, and P values were indicated, and error bars were shown as mean ± s.d. ^*^P< 0.05, ^**^P< 0.01, ^***^P< 0.001.

To determine whether PAC suppresses HCC growth through modulation of the gut microbiota, we first employed a subcutaneous xenograft model in mice. After 24 days of oral PAC administration (Fig. 1A), tumor volume and weight were markedly reduced compared with vehicle controls (P < 0.001; Fig. 1B~D), whereas body weight remained unchanged (Fig. 1E), indicating negligible systemic toxicity.

The therapeutic efficacy of PAC was corroborated in an orthotopic HCC model. PAC-treated mice exhibited a significant decrease in overall tumor burden (P < 0.001), fewer tumor nodules (P < 0.05), and a reduced maximal tumor diameter (P < 0.001; Fig. 1F~I), again without appreciable weight loss (Fig. 1J). Histopathological examination revealed a pronounced reduction in malignant cell density and diminished Ki67 staining in PAC-treated tumors (Fig. 1K). Importantly, no histological alterations were detected in the colon of treated mice (Fig. 1L), ruling out local gastrointestinal toxicity. At the molecular level, qRT-PCR showed robust up-regulation of Caspase-3 and P53 mRNA, together with down-regulation of cyclin D1, in PAC-treated tumors (Fig. 1M~P). Western blotting confirmed these findings, revealing elevated levels of Bax, cleaved caspase-3, and p53, and reduced cyclin D1 protein (Fig. 1Q). Collectively, these data demonstrate that PAC administration profoundly inhibits HCC progression by curbing proliferation and potentiating apoptosis, underscoring its potential as a gut-microbiota-targeted therapeutic agent for HCC.

### Procyanidin treatment upregulates the MAPK pathway in B cells within the tumor immune microenvironment of orthotopic liver cancer mice via the gut-liver axis

**Figure 2:**
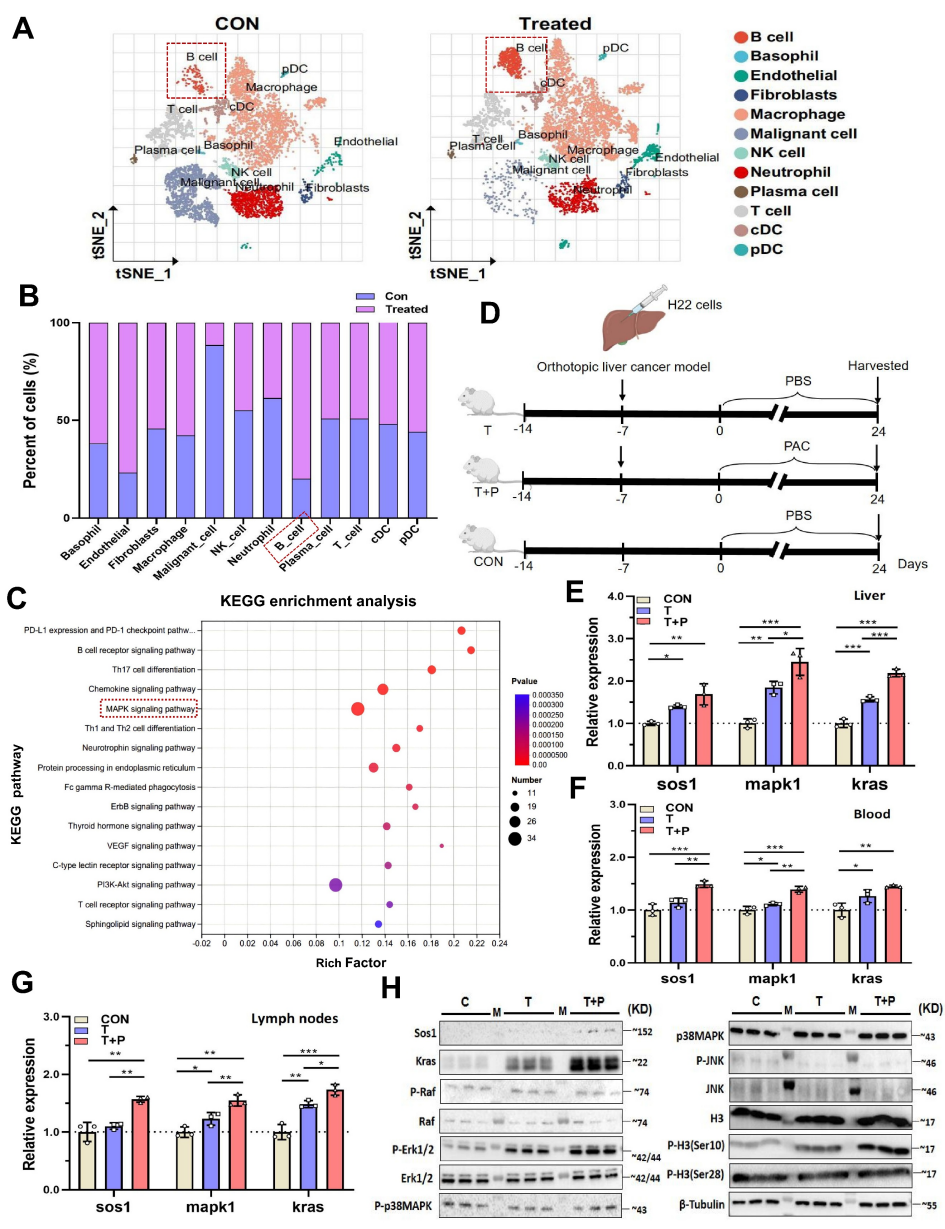
** Procyanidin treatment upregulates the MAPK pathway in B cells within the tumor immune microenvironment of orthotopic liver cancer mice via the gut-liver axis.** A. tSNE plot showing subcluster in tumor tissue on day 24 between the control group and the treated group in mice with orthotopic liver cancer. Tumors from three different mice were pooled per sample. B. Proportion chart of different cell subset. C. KEGG enrichment bubble chart of upregulated genes in B cells in the treated group. D. Schematic of the experiment in BALB/c mice with orthotopic liver cancer. E~G. Relative expression levels of different molecules in isolated B cells from MLNs, liver, and blood among the groups. H. Western blot images of different molecules in B cells from MLNs in each group. n=3 per group. All statistical data were presented as mean ± s.d, analyzed using t test. CON, tumor-free group. T, tumor-bearing mice group. T+P, tumor-bearing mice treated with PAC group. M, ladder. ^*^P < 0.05, ^**^P < 0.01, ^***^P < 0.001.

To investigate the tumor microenvironment (TME) of orthotopic liver cancer, we subjected murine liver tumors to single-cell RNA sequencing. After graph-based clustering and annotation with canonical markers, dimensionality reduction (t-SNE) resolved the main leukocyte compartments (Fig. 2A; Supplementary fig. 2A, B). Relative abundance testing identified B cells as the population that changed most markedly following therapy (Fig. 2B). KEGG enrichment of B-cell-restricted differentially expressed genes disclosed pronounced up-regulation of the MAPK signalling axis-specifically* Sos1, Kras* and *Mapk1*-in treated animals (Fig. 2C; Supplementary fig. 2C). Because nascent B-cell development is known to occur within the intestinal mucosa 12, 16, we reasoned that mucosal immune cues might re-programme the B-cell MAPK network and, secondarily, remodel the hepatic TME.

To test this hypothesis, B cells isolated from mesenteric lymph nodes (MLNs), liver and peripheral blood were used to quantify MAPK transcripts by RT-qPCR. HCC implantation already elevated* Sos1*,* Kras* and *Mapk1* expression, and PAC administration further amplified each transcript in all three anatomical compartments (Fig. 2D~G), mirroring the scRNA-seq signature. MAPKs govern gene expression by modulating mRNA turnover and nucleo-cytoplasmic shuttling 17 and can phosphorylate histone H3 at Ser10 and Ser28 18. We therefore asked whether PAC triggers this epigenetic module. Western blotting of MLNs revealed marked increases in SOS1, KRAS, phospho-ERK1/2 and phospho-H3(Ser10) within B cells (Fig. 2H). Together, these data implicate the gut-liver axis in PAC-mediated reprogramming of the B-cell MAPK pathway and, consequently, in reshaping the tumor microenvironment.

### PAC-induced alterations in gut microbiota are characterized by *Lactobacillus murinus*, and transplantation of *Lactobacillus murinus* significantly reduces tumor volume in mice with HCC

**Figure 3:**
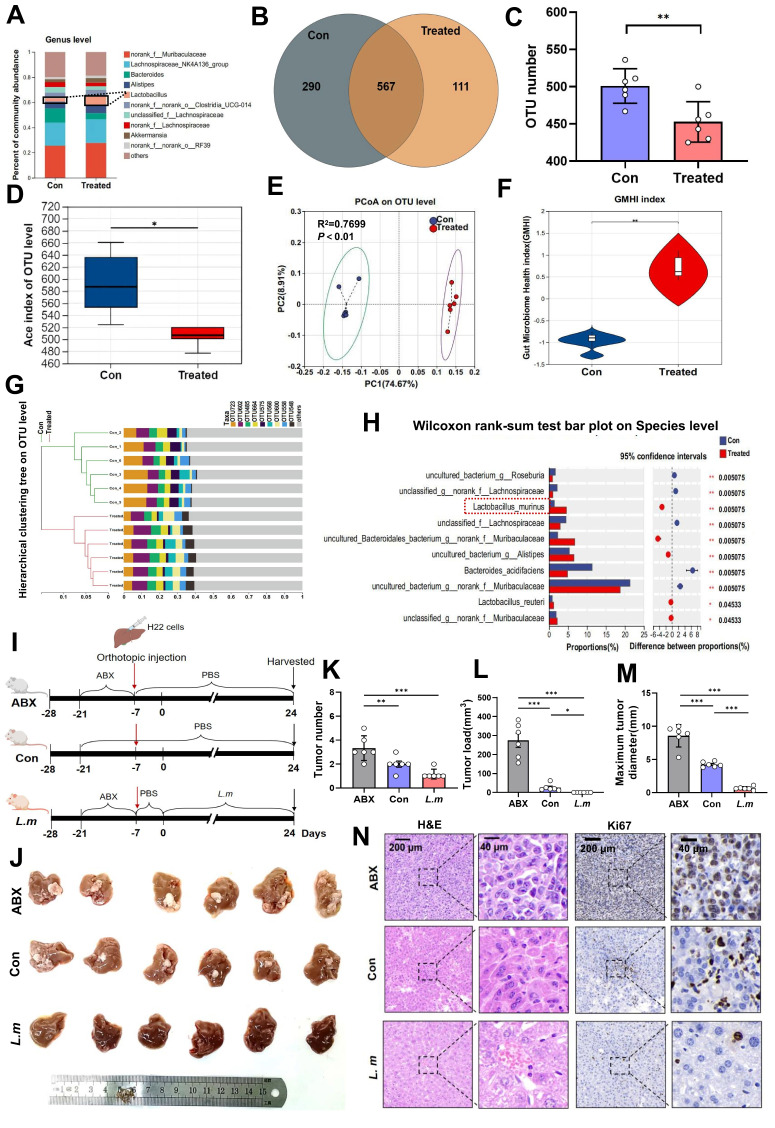
** Procyanidin can reshape gut microbiota in mice with orthotopic liver cancer.** A. Percentage of gut microbiota at the genus level between the PAC-treated group and the untreated group. B. Venn diagram of OTUs. C. Statistical chart of OTU number differences. D. The changes in ace index of OTU level. E. PCoA analyze on OTU level. F. Statistical chart of gut microbiota health index (GMHI). G. Hierarchical clustering tree at the OTU level. H. The differences in gut microbiota at the species level between the PAC-treated group and the untreated group. Significance was calculated using wilcoxon rank-sum test. I. Schematic of the experiment in BALB/c mice with orthotopic liver cancer. J. Images of liver tumors among the groups. K~M. Statistical charts of tumor number, load, and maximum tumor diameter. n=6 per group. N. H&E and Ki67 staining of tumors. The data were analyzed using t test. Significant P values were indicated, and error bars were shown as mean ± s.d. ^*^P < 0.05, ^**^P < 0.01.

Emerging evidence indicates that the intestinal microbiota shapes early B-cell development within the gut mucosa^19^. We therefore postulated that PAC remodels the luminal microbiome, thereby re-programming B-cell signalling in MLNs and, via the gut-liver axis, re-configuring the TME. Next, faecal pellets from tumor-bearing mice were subjected to 16S rDNA sequencing. Genus-, family- and phylum-level profiling, together with unsupervised heat-maps, disclosed marked compositional divergence between PAC-treated and control animals (Fig. 3A; Supplementary fig. 3A, B). At the OTU level, controls harboured 290 unique OTUs, whereas treated mice retained only 111 exclusive OTUs but shared 567 OTUs with controls (Fig. 3B; Supplementary fig. 3C), yielding a significant reduction in richness (P < 0.01, Fig. 3C). α-Diversity metrics confirmed lower overall diversity in the PAC group: Ace and Chao indices were both decreased (P < 0.05, Fig. 3D; Supplementary fig. 3D), whereas Simpsonevenness rose (P < 0.01; Supplementary fig. 3E). Coverage, Berger-Parker and inverse Simpson values remained equivalent, implying that PAC diminished richness while preserving evenness and sampling depth (Supplementary fig. 3F~H). β-Diversity analyses (NMDS: stress = 0.024, R² = 0.993; PCoA: R² = 0.770; PCA: R² = 0.731; all P < 0.01) demonstrated unambiguous separation between the groups (Fig. 3E; Supplementary fig. 3I, J). Consistently, the Gut Microbiome Health Index (GMHI) 20 was markedly elevated after PAC (P < 0.01, Fig. 3F), and hierarchical clustering of OTU profiles revealed coherent phylogenetic restructuring (Fig. 3G). LEfSe and LDA (> 2) highlighted a selective expansion of *L. murinus* as the dominant discriminator (Fig. 3H; Supplementary fig. 3M, N), while serum LPS concentrations fell (Supplementary fig. 4A). Collectively, PAC induces a less diverse but healthier microbiota characterised by* L. murinus* dominance and improved mucosal barrier function. To determine whether *L. murinus* itself restrains tumor outgrowth, mice were depleted of commensals with antibiotics (ABX) and subsequently gavaged with *L. murinus* (Fig. 3I). Compared with ABX-only animals, *L. murinus*-colonized mice exhibited profound reductions in tumor number, total tumor burden and maximum tumor diameter (P < 0.001), attaining values indistinguishable from untreated, conventionally colonized controls (Fig. 3J~N). Thus, *L. murinus* recapitulates a sizeable component of PAC-induced tumor control, corroborating the concept that microbiome-directed immune modulation along the gut-liver axis constrains HCC progression.

### The supernatant of *Lactobacillus murinus* mediates the B cell MAPK pathway and activates CD8^+^T cells *in vitro*

**Figure 4:**
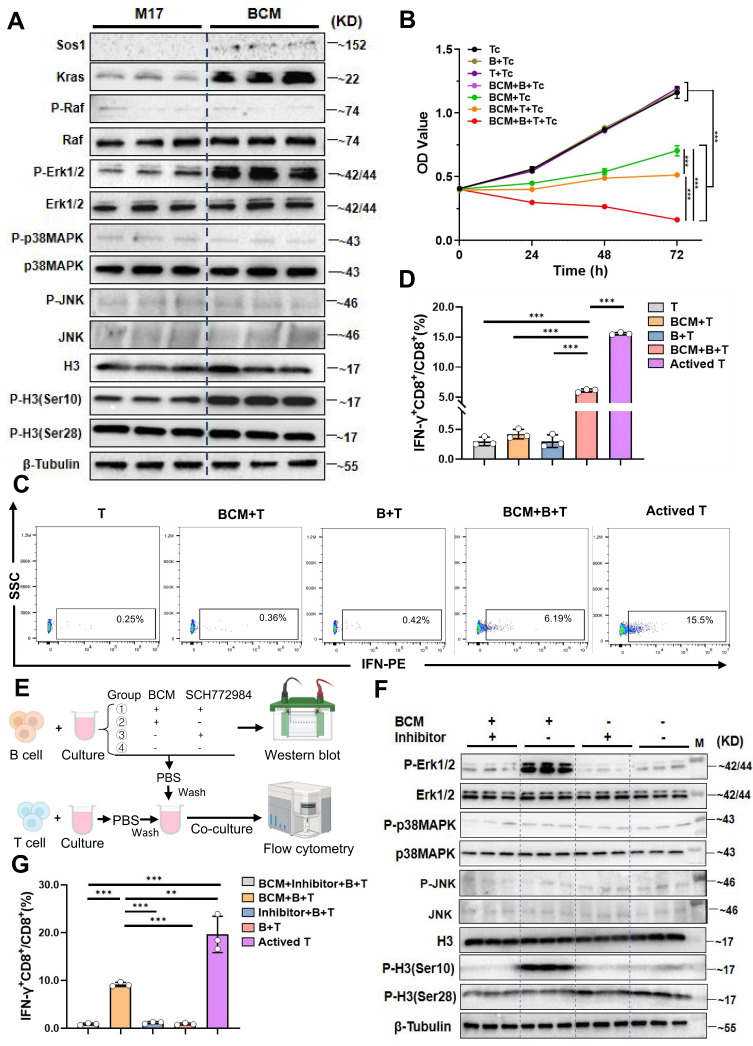
***Lactobacillus murinus* supernatant mediates the MAPK pathway in B cells and activates CD8^+^T cells.** A. Western blot validation of key proteins in the MAPK pathway after culturing B cells with 7.5% BCM for 24 hours. B. *In vitro* proliferation experiment of B cells treated with 7.5% BCM, T cells, and tumor cells. C. Flow cytometry analysis of the percentage of IFN-γ^+^CD8^+^/CD8^+^T cells among the groups. D. Statistical chart of IFN-γ^+^CD8^+^/CD8^+^T cells. E. Schematic of the experimental steps where BCM educated B cells were co-cultured with T cells. F. Western blot analysis of various proteins in the MAPK pathway. M, ladder. G. Flow cytometry was used to measure the percentage of IFN-γ^+^CD8^+^/CD8^+^T cells. n=3 per group. All data were analyzed using the t test, and statistical data are presented as mean ± s.d. ^**^P < 0.01, ^***^P < 0.001.

Studies have shown that microbial metabolites can directly modulate immune-cell signalling 21. To ask whether *L. murinus*-derived factors engage the B-cell MAPK axis, we exposed purified B cells to serial dilutions of* L. murinus*-conditioned medium (BCM). A concentration of 7.5 % (v/v) elicited robust phosphorylation of ERK1/2 and histone H3(Ser10) within 24 h (P < 0.001; Supplementary Fig. 4F~H); expression of Sos1 and Kras was also increased relative to control M17 medium (Fig. 4A). Tumor-cell lines exposed to identical BCM remained unresponsive, confirming specificity for B cells (Supplementary Fig. 4O).

Microbial products can activate resting B cells via Toll-like receptors or the B-cell receptor, thereby enhancing antigen presentation to T cells 22. Classic cellular immunity suggests that the interaction between B cells and T cells includes promoting antigen-specific T cell responses, enhancing memory immune responses, and regulating the TME to suppress tumor growth and metastasis 23. We therefore asked whether BCM-licensed B cells promote cytotoxic T-cell immunity. Humoral immunity was first excluded: serum IgA and IgG value did not differ between control and PAC-treated groups (Supplementary Fig. 4I, J), and BCM stimulation did not increase antibody secretion *in vitro* (Supplementary Fig. 4M, N). Mono-colonization with *L. murinus* significantly elevated systemic IgA levels (Supplementary fig. 4K, L), implying that the bacterium itself-rather than global microbiota remodeling-drives mucosal IgA. To examine cellular immunity, BCM-educated B cells were co-cultured with CD8⁺T cells and Hepa1-6 cells. Tumor proliferation was lowest in the triple-culture condition (P < 0.001, Fig. 4B), accompanied by a selective expansion of IFN-γ⁺CD8⁺T cells (P < 0.001 versus B-T, BCM-T or T alone; Fig. 4C, D). Pharmacological blockade of ERK with SCH772984 abolished both MAPK phosphorylation in B cells (Fig. 4E, F) and the subsequent increase in IFN-γ⁺CD8⁺ T cells (P < 0.001, Fig. 4G; Supplementary Fig. 5B). Thus, a soluble *L. murinus* product activates the B-cell MAPK pathway and licenses CD8⁺T-cell cytotoxicity.

### The metabolite 5-hydroxytryptophan from *Lactobacillus murinus* upregulates the MAPK pathway in B cells within mesenteric lymph nodes and activates CD8^+^T cells in the tumor immune microenvironment

**Figure 5:**
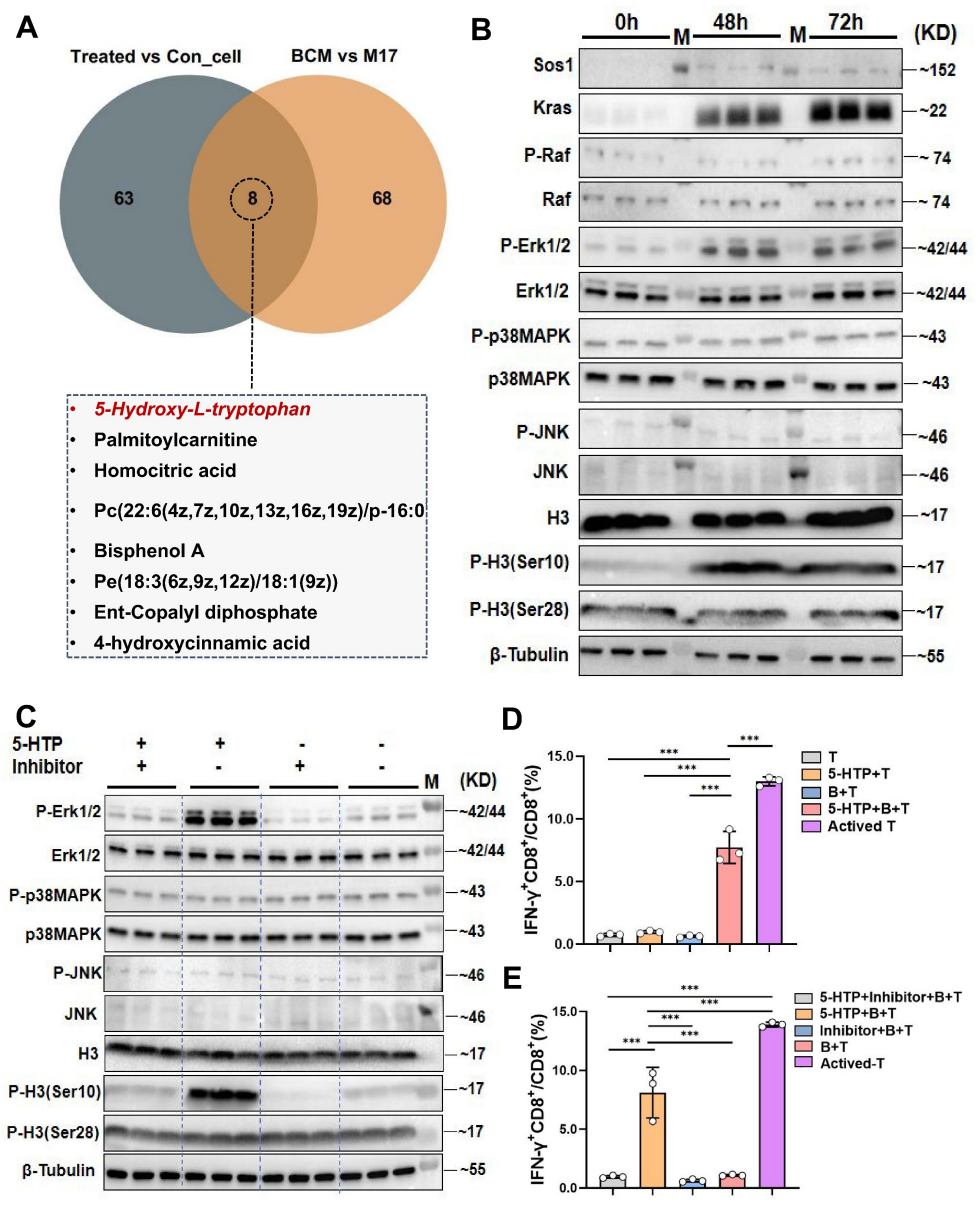
***Lactobacillus murinus* related metabolite 5-HTP activates the MAPK pathway in B cells and interacts with T cells to activate CD8^+^T cells.** A. Venn diagram of differential metabolites in MLNs B cells from the treated group and control group and metabolomic analysis of BCM and M17. B. Western blot analysis after educating B cells with 5-HTP. C. Western blot analysis of various proteins in the MAPK pathway. D. cytometry was used to measure the IFN-γ^+^CD8^+^/CD8^+^T cells (%). E. Flow cytometry was used to measure the IFN-γ^+^CD8^+^/CD8^+^T cells (%). n=3 per group. M, ladder. All data were analyzed using the t test, and statistical data were presented as mean ± s.d. ^***^P < 0.001.

To identify the bioactive metabolite(s) through which *L. murinus* “educates” B cells, we performed untargeted metabolomics on MLN B cells from PAC-treated and control tumor-bearing mice, and on cells stimulated with BCM or M17 medium. Seventy-one metabolites were enriched in B cells from treated mice and 76 in the BCM group; intersection revealed eight candidates common to both datasets (Fig. 5A; Supplementary fig. 5C, D). Among these, 5-hydroxytryptophan (5-HTP)-the immediate precursor of serotonin-stood out. Tryptophan is largely supplied by diet and microbial catabolism, and its conversion to 5-HTP, kynurenine or indole derivatives is governed by the gut microbiota 24, 25. We therefore asked whether 5-HTP produced by *L. murinus* might be the critical signal. Although serum 5-HTP was unchanged, faecal levels were significantly higher in PAC-treated mice than in untreated HCC controls; both compartments were depleted in antibiotic-treated animals, consistent with a microbial origin (Supplementary fig. 2B~E). *In vitro*, 10~40 µM 5-HTP promoted B-cell proliferation after 48~72 h (P < 0.001; Supplementary fig. 5E). At 10 µM, 5-HTP recapitulated the BCM signature: up-regulation of SOS1, KRAS, phospho-ERK1/2 and phospho-H3(Ser10, Fig. 5B). ERK inhibition with SCH772984 abolished these changes (Fig. 5C), confirming MAPK dependence.

**Figure 6:**
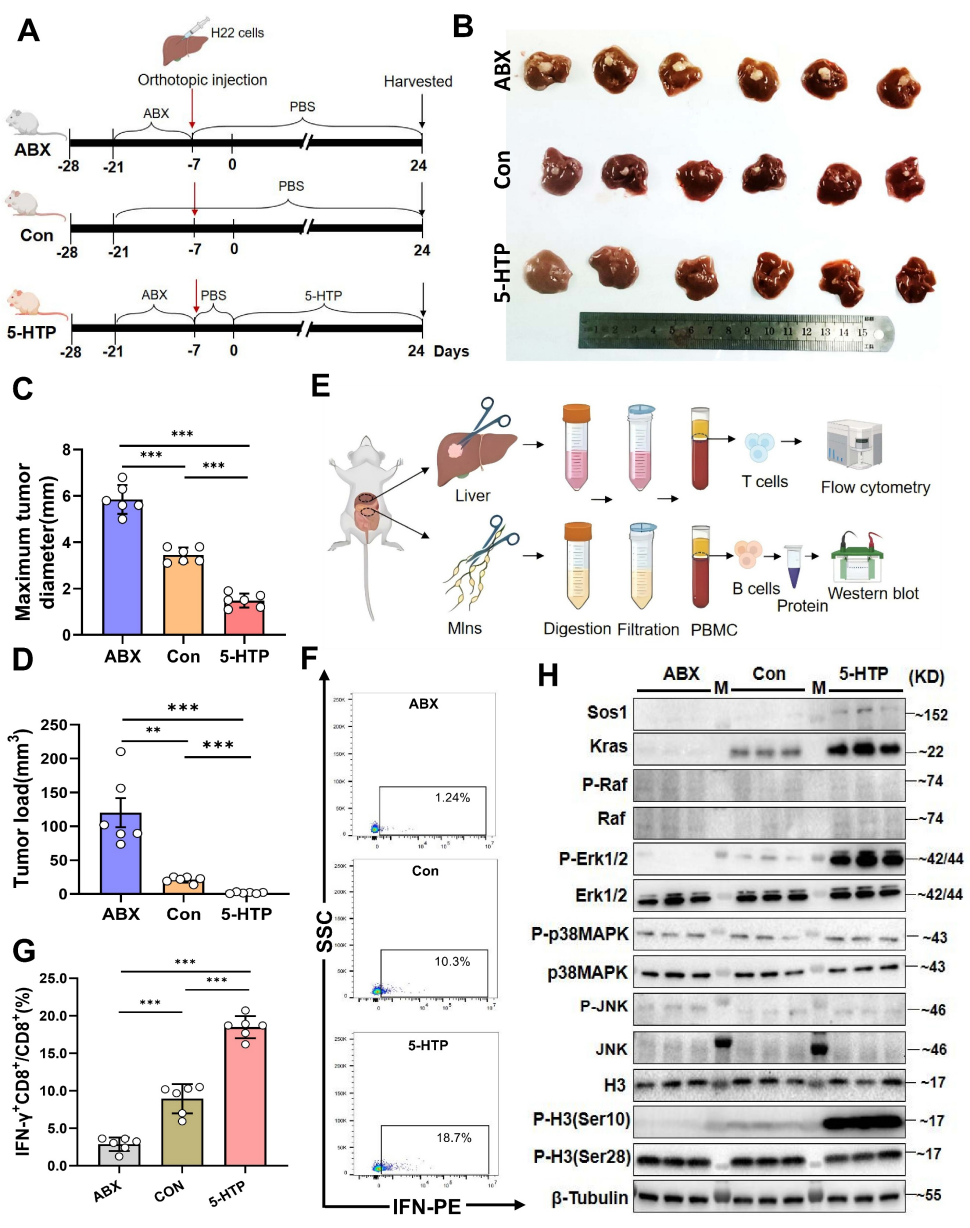
** 5-HTP induces a reduction in liver cancer volume in mice, leading to activation of MAPK pathway molecules in B cells in MLNs and activation of IFN-γ^+^CD8^+^T cells in the tumor immune microenvironment.** A. Schematic of the experiment conducted in BALB/c mice with orthotopic liver cancer. B. Photographs of subcutaneous tumors in mice among the groups. C. Bar graph of maximum tumor diameter. D. Bar graph of tumor load. E. Diagram of experimental steps for extracting B and T cells from MLNs and tumor tissue for flow cytometry and western blot validation. F, G. Percentages of IFN-γ^+^CD8^+^/CD8^+^T cells in TME among the groups. H. Western blot results of various proteins in B cells from the MLNs among groups. n=6 per group. M, ladder. All data were analyzed using the t test, and statistical data were presented as mean ± s.d. ^**^P < 0.01, ^***^P < 0.001.

Functionally, 5-HTP-primed B cells co-cultured with CD8⁺T cells and Hepa1-6 cells triggered robust IFN-γ production and suppressed tumor growth (P < 0.001 versus all controls; Fig. 5D; Supplementary fig. 5F). SCH772984 again abrogated cytotoxicity (P < 0.001; Fig. 5E; Supplementary fig. 5G). Finally, daily 5-HTP administration to orthotopic HCC mice reduced tumor burden and maximal diameter (P < 0.001; Fig. 6A~D), coincident with MAPK activation in MLN B cells (Fig. 6E, H) and a marked expansion of IFN-γ⁺CD8⁺ T cells within the TME (P < 0.001; Fig. 6F, G). Thus, 5-HTP-metabolite enriched when *L. murinus* dominates the PAC-reshaped microbiota-suffices to ignite the B-cell MAPK axis, licence CD8⁺T-cell immunity and restrain HCC.

## Discussion

Currently, most research on PAC has focused on their direct effects on tumor cells, while limited knowledge exists regarding their immunological mechanisms against HCC. Numerous studies have demonstrated that procyanidins exert health-promoting effects through their immunomodulatory properties in the gut 26. Additionally, research has shown that PAC can effectively prevent colorectal cancer by interacting with both the host and microbial communities 27. The gut microbiota plays a crucial role in regulating host immune function, and emerging evidence suggests that the interaction between PAC and the gut microbiota significantly influences human health. However, current studies on PAC-mediated reshaping of the gut microbiome are primarily centered on metabolic syndrome and cardiovascular diseases, with limited investigation into their effects on the gut microbiota in the context of HCC 28. Further research is warranted to elucidate how PAC-induced alterations in the gut microbiota and host interactions contribute to their potential protective effects against HCC. 16S rDNA sequencing revealed a significant increase in *L. murinus* in the treated group. Studies have confirmed that PAC, by interacting with the gut microbiota, exerts prebiotic-like effects that enrich beneficial taxa such as *Lactobacillus* and *Bifidobacterium* 28. This finding aligns with our observation of reduced tumor volume in the treated group. Research has shown that *Lactobacilli* enhances barrier function by regulating luminal pH, increasing mucus production, secreting antimicrobial peptides, altering gut microbiota composition, competing with opportunistic pathogens, participating in early immune system maturation, maintaining immune homeostasis, and producing metabolites, vitamins, and other components 29. The role of gut microbiota in gastrointestinal-related cancers is a double-edged sword. On one hand, its protective functions include regulating the immune system, maintaining intestinal barrier integrity, and metabolizing anti-carcinogenic compounds^30^, ^31^. On the other hand, its pathogenic aspects can promote inflammatory responses, exacerbate the tumor microenvironment, and facilitate immune evasion. Furthermore, dysbiosis of the gut microbiota has profound impacts on cancer initiation, progression, and response to treatment. Therefore, elucidating the underlying mechanisms of gut microbiota function and restoring microbial balance hold significant clinical value for the prevention and treatment of cancer^32^. It has been demonstrated that co-administration of *Lactobacillus reuteri* and PD-L1 inhibitors significantly enhances the anti-tumor effects 33. Therefore, we hypothesize that *L. murinus* may be the key microbiota reshaped by PAC that triggers host immunity, leading to tumor reduction.

Single-cell RNA-seq revealed that treatment reduced malignant cells and enriched B cells within the TME. Because B-cell development occurs in the intestinal lamina propria (LP), we next asked whether the gut microbiota reshapes the B-cell receptor (BCR) repertoire at this site. Colonization of germ-free (GF) mice with a conventional microbiota during weaning increased the frequency of RAG2⁺B cells in the LP relative to age-matched GF controls 16, indicating that microbial signals promote intestinal B-cell differentiation. The molecular underpinnings of this microbiota-driven B-cell education remain to be defined. KEGG profiling of B-cell transcripts identified a selective enrichment of the MAPK cascade in PAC-treated tumors, driven by up-regulation of Sos1, Kras and Erk. MAPKs-evolutionarily conserved Ser/Thr kinases-integrate mitogenic, stress and immune cues to control proliferation, survival and differentiation. In mammals, the canonical RAS-RAF-MEK-ERK axis and the p38 (MAPK14) module transduce signals initiated by diverse surface receptors. Within the B-lineage, RAS-MAPK activity is indispensable for early development, antigen-dependent expansion and long-term survival, functioning as the immediate downstream output of BCR engagement 17, 34-36. Expansion of *L. murinus* after PAC administration coincided with MAPK activation in intratumoral B cells, prompting the hypothesis that this microbe licenses B-cell expansion via the gut-liver axis. In an HCC model, gavage with *L. murinus* alone recapitulated the tumor-suppressive effect observed in PAC-treated mice. Mechanistically, activated MAPKs phosphorylate transcription factors and remodel chromatin-ERK-mediated phosphorylation of histone H3 at Ser10 and Ser28 is a well-documented example 17, 18, 37, 38. Flow cytometric analysis of B cells harvested from blood, liver and MLNs confirmed PAC-induced up-regulation of Sos1, Kras and phospho-ERK1/2, together with elevated H3-S10ph in MLNs. Collectively, PAC appears to trigger *L. murinus*-dependent MAPK signaling that propagates from the intestinal lamina propria to the tumor bed, thereby potentiating anti-tumor B-cell immunity. PAC-driven enrichment of *L. murinus* prompted us to ask whether its metabolites, rather than systemic humoral immunity, underlie MAPK-dependent B-cell licensing. *Lactobacillus*-derived metabolites are already known to potentiate immune-checkpoint blockade; e.g., *Lactobacillus plantarum* restrains colitis-associated tumorigenesis via arachidonic-acid reprogramming and CD22-mediated BCR tuning 39, 40. Accordingly, B cells cultured with *L. murinus*-conditioned medium recapitulated the MAPK signature observed in MLN B cells from PAC-treated mice: up-regulation of Sos1, Kras, phospho-ERK1/2 and phospho-H3(S10). To test functional output, BCM-educated B cells were co-cultured with splenic T cells. Tumor-cell killing was markedly enhanced, and flow cytometry showed a rise in IFN-γ⁺CD8⁺/CD8⁺T cells ratio, indicating that metabolite-primed B cells licence cytotoxic T-cell immunity. Single-cell interactome analysis revealed strengthened ALCAM-CD6 pairing between B cells and Tc1 cells after PAC exposure (Supplemental Fig. 2D), a contact known to stabilise the immunological synapse and amplify TCR signalling 41, 42. Requirement for MAPK activity was confirmed pharmacologically: the ERK inhibitor SCH772984 abolished BCM-induced phospho-ERK1/2 in B cells and concurrently reversed the increase in IFN-γ⁺CD8⁺T cells. Thus, *L. murinus* metabolites activate the B-cell MAPK axis to propagate CD8⁺T-cell immunity against HCC. *In vitro*, 5-HTP reproduced the full BCM phenotype: Sos1/Kras/phospho-ERK1/2 induction in B cells and downstream expansion of IFN-γ⁺CD8⁺ T cells; both responses were abrogated by ERK inhibition. 5-HTP is a microbially converted tryptophan metabolite and rate-limiting precursor of 5-hydroxytryptamine (5-HT), a neurotransmitter increasingly recognized as an immune modulator at mucosal and systemic levels 4, 43. The present data extend this paradigm by demonstrating that a single microbiota-derived 5-HTP cue is sufficient to couple B-cell MAPK signalling to cytotoxic T-cell output, consolidating the host-microbiota-serotonin axis as a therapeutic checkpoint in HCC.

**Figure 7:**
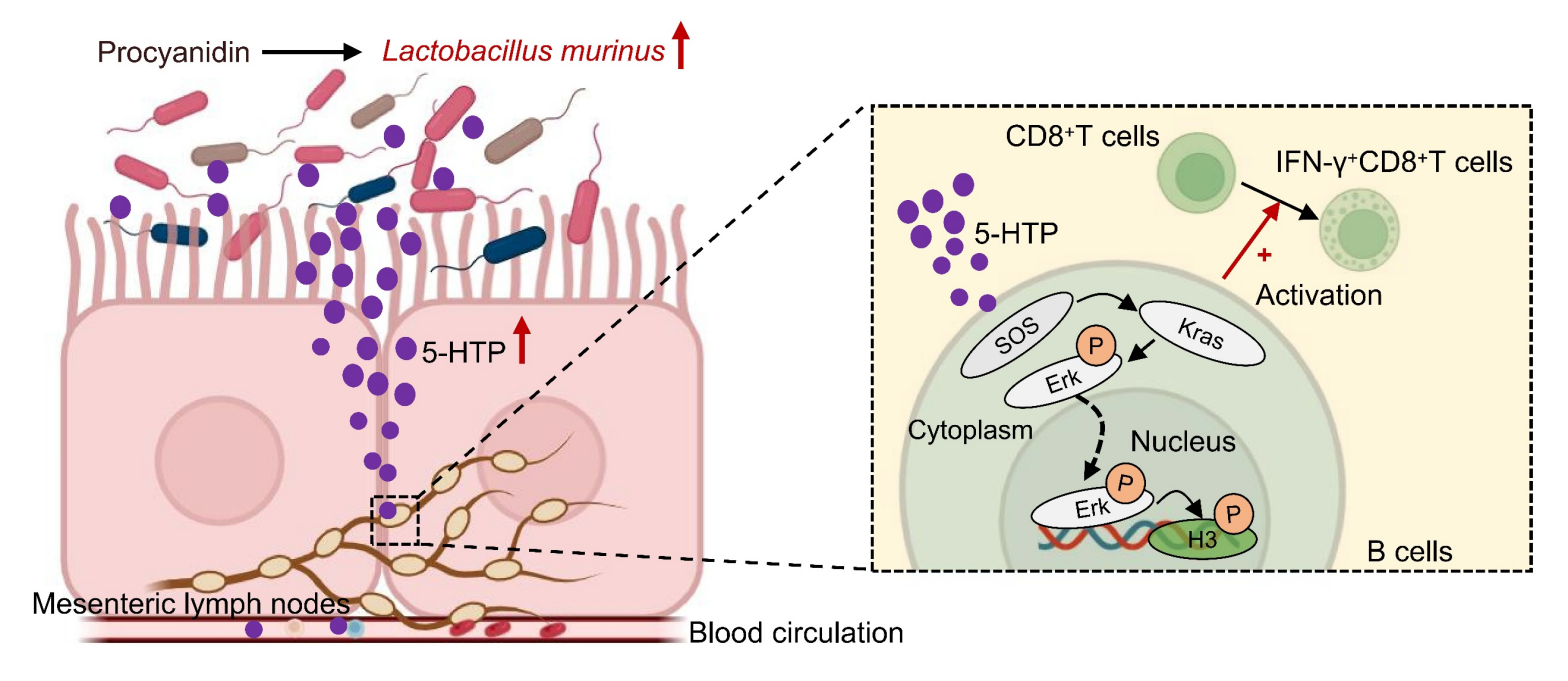
** Hypothetical mechanism by which procyanidin suppresses the tumor growth of HCC by altering gut microbiota in the host.** After treatment with procyanidin in mice with HCC, the abundance of *Lactobacillus murinus* was increased in gut, leading to the upregulation of Sos1, Kras, and phospho-Erk1/2 in the MAPK pathway in B cells mediated by the associated metabolite 5-HTP. phospho-Erk1/2 may act as a transcription factor binding to promoters, causing changes at the S10 site of histone H3, and activating IFN-γ^+^CD8^+^T cells, thereby exerting anti-tumor effects.

In summary, PAC rewires the gut-tumor axis in HCC: microbiota expansion of *L. murinus* enriches 5-HTP, which triggers MAPK signalling in mesenteric-lymph-node B cells, thereby licensing IFN-γ⁺CD8⁺T cells and suppressing tumor growth (Fig. 7). The study is limited to pre-clinical models and awaits clinical validation; moreover, PAC-induced microbiota changes may engage additional, yet-to-be-defined immunoregulatory circuits. This study redefines the immunomodulatory repertoire of PAC by uncovering a microbiota-dependent, gut-liver axis that licenses B-cell-driven CD8⁺T-cell immunity against hepatocellular carcinoma. As safe, low-cost dietary polyphenols, PAC can be readily translated into adjuvant regimens to reinforce standard therapies and offer patients a non-invasive dietary leverage for improved outcomes.

## Supplementary Material

Supplementary figures.
