# Supplementary material for: Procyanidin Suppresses Tumor Growth by Activating the B-Cell MAPK Pathway through Remodulation of the Gut Microbiota and Metabolites in Hepatocellular Carcinoma
Source: Int J Biol Sci. 2026 Jan 1;22(1):161–77. doi: 10.7150/ijbs.113217 (PMC12681853; doi:10.7150/ijbs.113217)
Supplement: Supplementary file 1 — Supplementary figures. [file ijbsv22p0161s1.pdf]

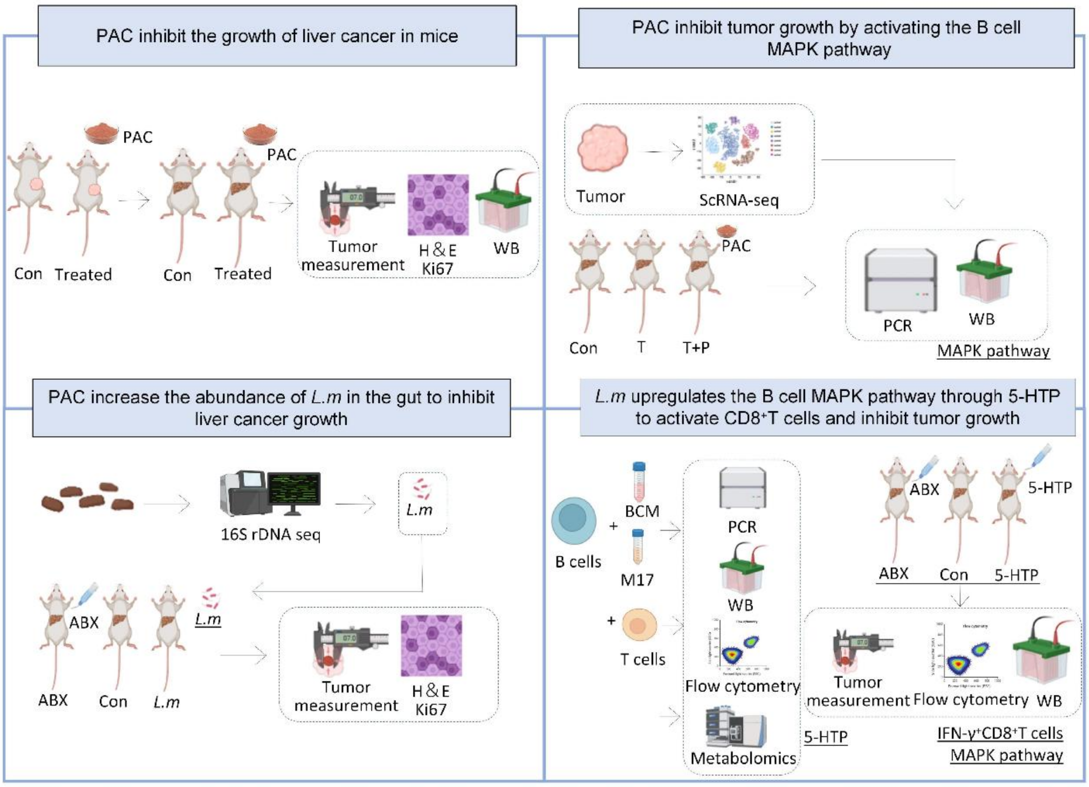

Supplemental fig.1. A scheme on the design of the study.

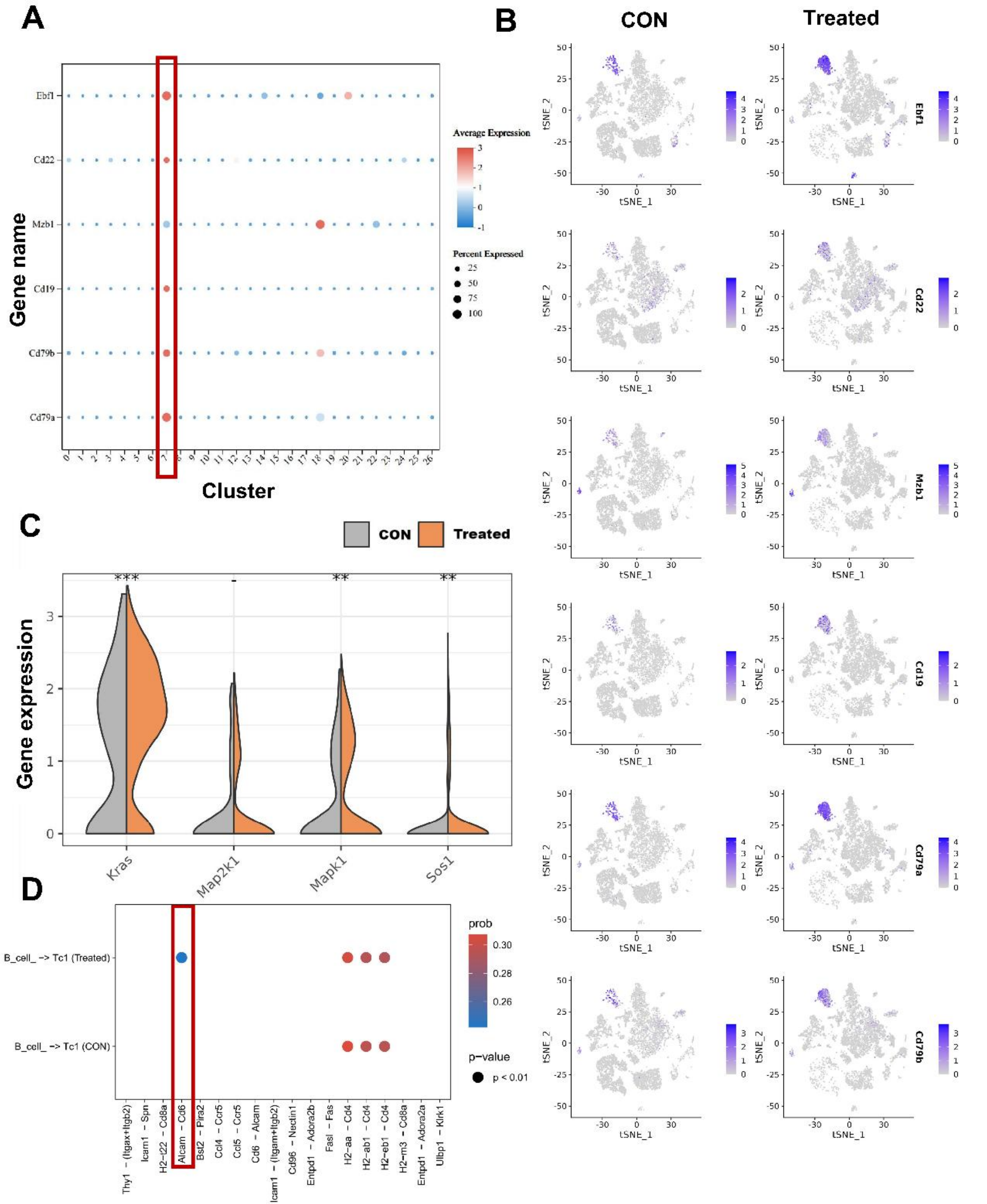

Supplemental fig.2. ScRNA-seq analysis between the two groups

A. B cell marker expression bubble chart of the 7-cluster. B. tSNE plots of marker expression. C. Statistical analysis of upregulated gene expression in the MAPK pathway. D. Analysis of receptor-ligand interactions between B cells and T cells. Tumors from three different mice were pooled per sample. $^{*}P < 0.05$, $^{**}P < 0.01$, $^{***}P <$

0.001.

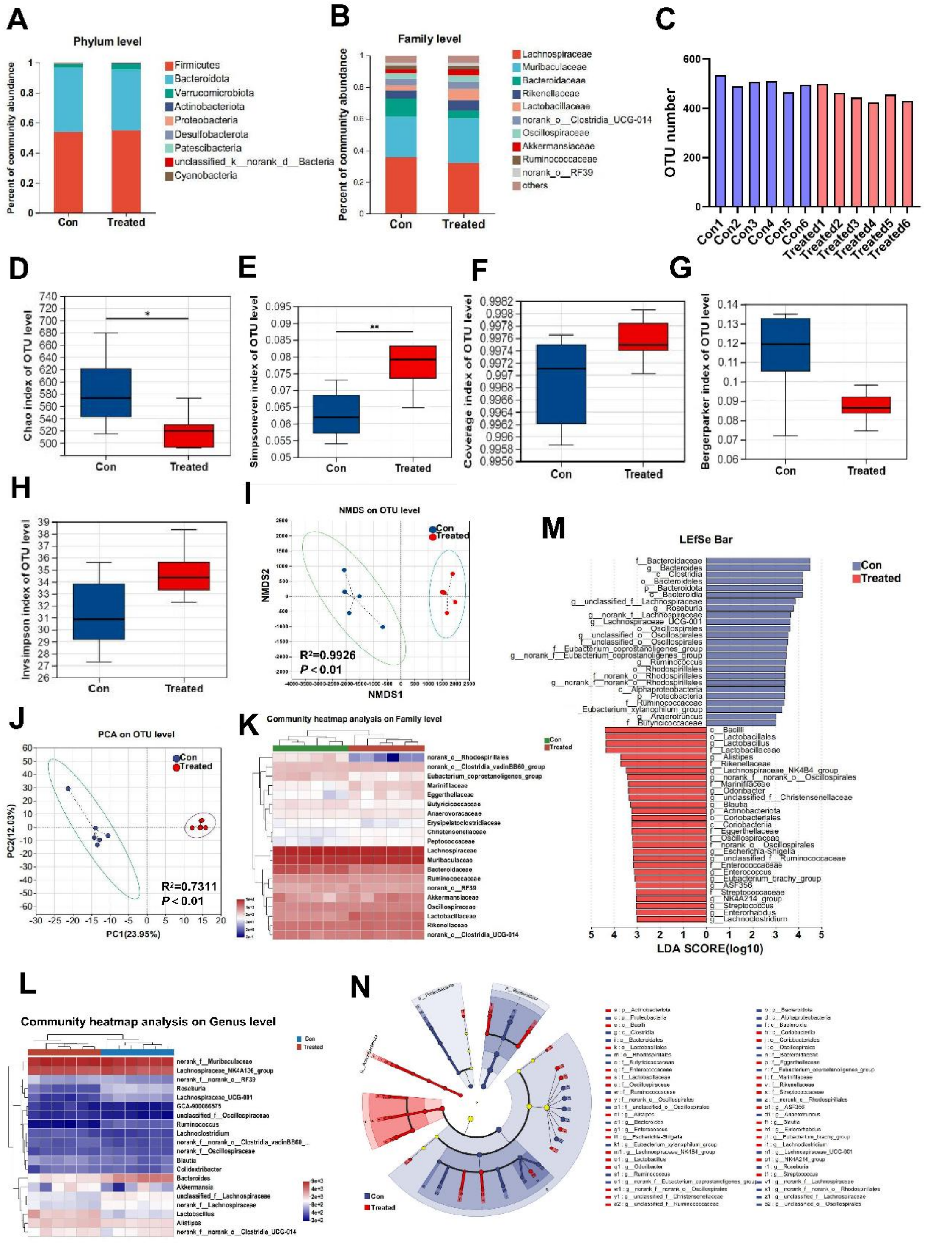

Supplemental fig.3. Analysis of 16S rDNA sequencing results

A, B. Percentages of gut microbiota at the phylum (A) or family (B) level. C. Bar chart showing the number of OTUs. D~H. Analysis of chao (D), simpsoneven (E),

coverage (F), bergerparker (G), and invsimpson (H) index. I, J. Statistical charts of NMDS (I) and PCA (J) indices. K, L. Clustering heatmap on family (K) and genus (L) level. M. LEfSe hierarchical tree from phylum to genus level. N. Linear discriminant analysis (LDA > 2) from phylum to genus level between the two groups. n=6 per group. All data were analyzed using the t test, and statistical data were presented as mean±s.d.$^{*}P < 0.05$， $^{**}P < 0.01$.

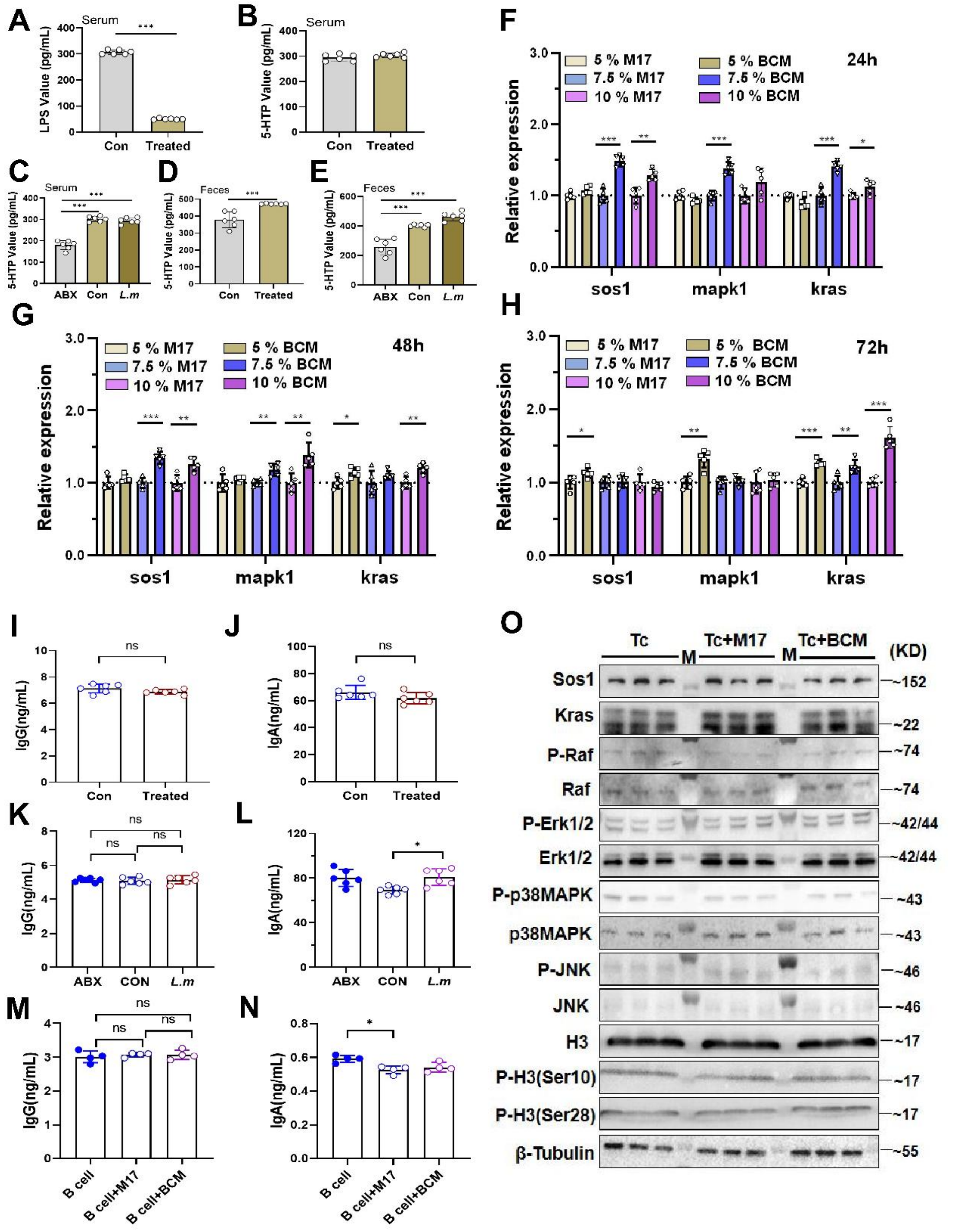

Supplemental fig.4. Experimental Results of RT-qPCR, Western Blot, and ELISA among the groups

A. Statistical analysis of serum LPS levels between the treatment and control groups. B~E. Statistical analysis of serum and fecal 5-HTP levels among different groups. n = 6 per group. F~H. Expression of sos1, mapk1, and kras in B cells after culturing with

5%, 7.5%, and 10% BCM or M17 for 24 (F), 48 (G), and 72h (H). n=5 per group. I~J. The changes in IgG and IgA levels in serum among the control group and treated. K~L. The changes in IgG and IgA levels in serum among the ABX, Con, and *L.m* groups. n=6 per group. M~N. The changes in IgG and IgA levels in the culture solution among the B, M17-B, and BCM-T groups. n=4 per group. O. The changes in MAPK pathway proteins in tumor cells cultured with 7.5% BCM or 7.5% M17 for 24 hours. n=3 per group. M, ladder. All data were analyzed using the t test, and statistical data were presented as mean ± s.d. ns, $P > 0.05$, $^{*}P < 0.05$, $^{**}P < 0.01$, $^{***}P < 0.001$.

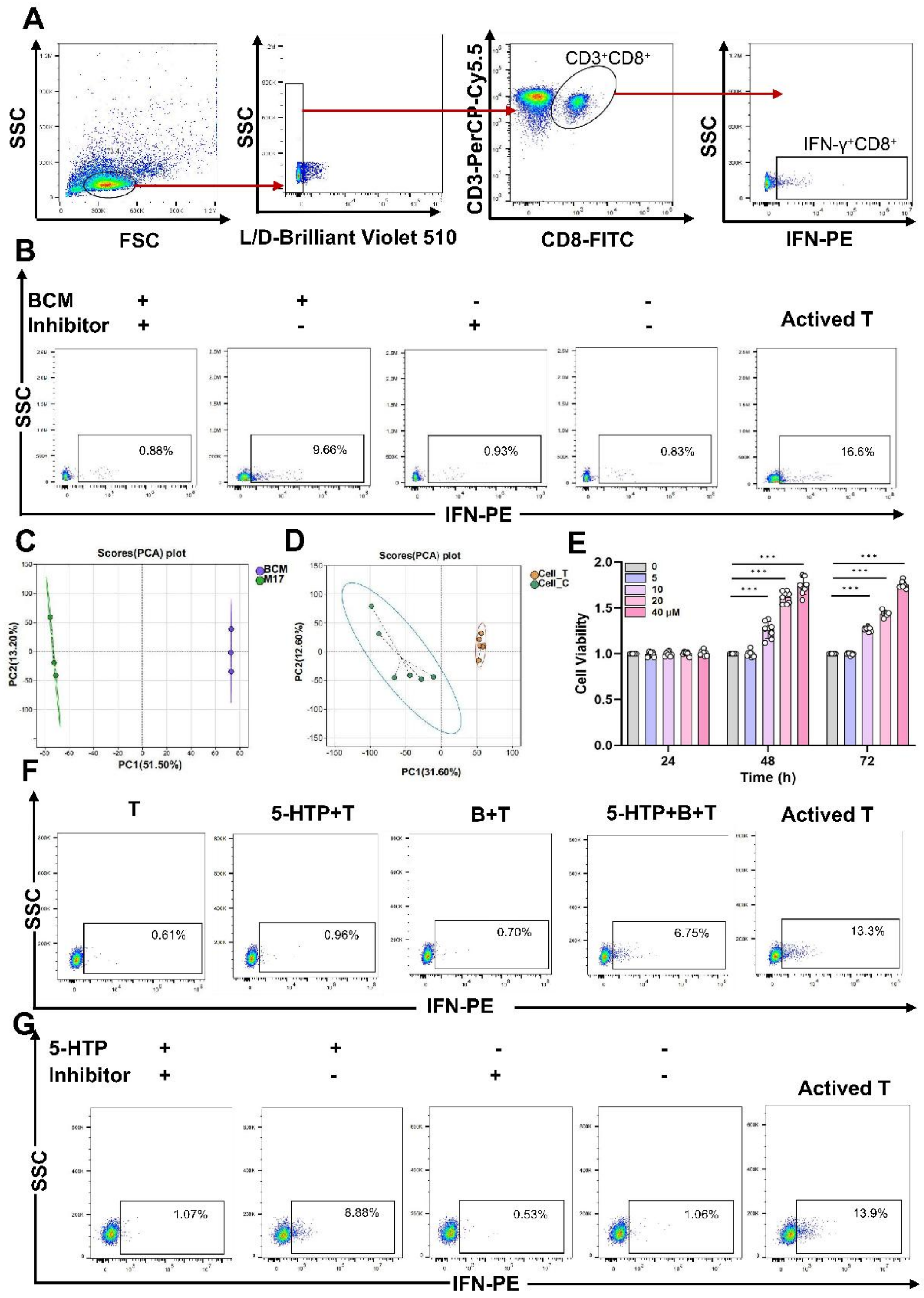

Supplemental fig.5. Flow cytometry experiment and metabolite analysis among the groups

A. Gating strategy for flow cytometry measurement of IFN-γ^{+}CD8^{+}/CD8^{+}T cells (%).

B. IFN-γ^{+}CD8^{+}/CD8^{+}T cells (%) was detected using flow cytometer. C. PCA analysis

of metabolites between the BCM and M17 groups, n=3 per group. D. PCA analysis of metabolites from MLNs B cells between the treated and control groups. n=6 per group. E. CCK-8 assay results after culturing B cells with 5-HTP. n=8 per group. F~G. IFN-γ^{+}CD8^{+}/CD8^{+}T cells (%) was detected using flow cytometer. n=3 per group. All data were analyzed using the t test, and statistical data were presented as mean ± s.d. $^{***}P < 0.001$.
